# Laparoscopic inguinal hernia repair: a prospective evaluation at Eastern Nepal

**DOI:** 10.11604/pamj.2014.17.241.2610

**Published:** 2014-03-29

**Authors:** Vikal Chandra Shakya, Shasank Sood, Bal Krishna Bhattarai, Chandra Shekhar Agrawal, Shailesh Adhikary

**Affiliations:** 1 Department of Surgery, B. P. Koirala Institute of Health Sciences, Dharan, Nepal; 2 Department of Anesthesia, B. P. Koirala Institute of Health Sciences, Dharan, Nepal

**Keywords:** Extraperitoneal repair, transabdominal approach, seroma, inguinal hernia, laparoscopy

## Abstract

**Introduction:**

Inguinal hernias have been treated traditionally with open methods of herniorrhaphy or hernioplasty. But the trends have changed in the last decade with the introduction of minimal access surgery.

**Methods:**

This study was a prospective descriptive study in patients presenting to Surgery Department of B. P. Koirala Institute of Health Sciences, Dharan, Nepal with reducible inguinal hernias from January 2011 to June 2012. All patients >18 years of age presenting with inguinal hernias were given the choice of laparoscopic repair or open repair. Those who opted for laparoscopic repair were included in the study.

**Results:**

There were 50 patients, age ranged from 18 to 71 years with 34 being median age at presentation. In 41 patients, totally extraperitoneal repair was attempted. Of these, 2 (4%) repairs were converted to transabdominal repair and 2 to open mesh repair (4%). In 9 patients, transabdominal repair was done. The median total hospital stay was 4 days (range 3-32 days), the mean postoperative stay was 3.38±3.14 days (range 2-23 days), average time taken for full ambulation postoperatively was 2.05±1.39 days (range 1-10 days), and median time taken to return for normal activity was 5 days (range 2-50 days). One patient developed recurrence (2%). None of the patients who had laparoscopic repair completed complained of neuralgias in the follow-up.

**Conclusion:**

Laparoscopic repair of inguinal hernias could be contemplated safely both via totally extra peritoneal as well as transperitoneal route even in our setup of a developing country with modifications.

## Introduction

Inguinal hernia repair is one of common general surgical operations [1]. Inguinal hernias have been treated traditionally with open methods of herniorrhaphy or hernioplasty. But the trends have changed in the last decade with the introduction of minimal access surgery. Laparoscopic inguinal hernia repair have been introduced after the success of laparoscopic cholecystectomy on the premise that there would be less postoperative discomfort and pain, the repair of recurrent hernias would be easier, and bilateral hernia could be treated concurrently with improved cosmesis [2]. Ger first reported laparoscopic inguinal hernia repair [3]. The first method used for this had been the transabdominal pre-peritoneal (TAPP) approach, but issues such as violation of the peritoneal cavity and occurrence of several complications, e.g, intestinal obstruction subsequent to entry of the peritoneal cavity had always been a concern for this approach [3]. Slowly another laparoscopic technique became widely used: the total extra-peritoneal (TEP) procedure because TEP repair is still considered to be an “advanced” laparoscopic procedure because of the unfamiliar anatomy and requires considerable training and laparoscopic experience [4]. Laparoscopic hernia repair has been introduced newly in our hospital. It is high time to move forward in laparoscopic techniques; repair of inguinal hernias would be the next step after cholecystectomies. Most of the studies are from the western world, which does not reflect the true picture from developing countries like ours. Doubt can be aroused whether these procedures can have a place in our set up, so it was a high time that such a kind of study be conducted to find out the efficacy of minimal access surgery in region and setup in a developing country like ours.

## Methods

This study was a prospective descriptive study in patients presenting to Surgery Department of B. P. Koirala Institute of Health Sciences, Dharan, Nepal with a diagnosis of reducible inguinal hernias from January 2011 to June 2012. All patients >18 years of age presenting with inguinal hernias were given the option of undergoing open or laparoscopic repair. Those who opted for laparoscopic repair were included in the study:

**Inclusion criterion**: elective hernia repair with age >18 years choosing laparoscopic repair. **Exclusion criteria**: patients were excluded if they opted for open repair, presence of complicated hernia, such as obstructed or strangulated hernia, uncorrectable coagulopathy, pregnant females, and patients unfit for general anesthesia.

All of the patients were included in the study after informed written consent. The study was conducted in accordance with the humane and ethical principles of research set forth in the Helsinki guidelines. The patients presenting with an inguinal/inguinoscrotal swelling were assessed thoroughly by clinical examination. History was taken regarding duration of the swelling, any association with features like pain, constipation, abdominal distension, vomiting, change in the size of swelling in supine or erect posture or straining. Physical examination was done and pulse, blood pressure, co-morbid conditions and inguinoscrotal examination including size, surface, cough impulse, reducibility, and fluctuation was noted. Once the clinical diagnosis of an uncomplicated inguinal hernia has been made, all the patients were explained in the native language about the laparoscopic procedure and an informed written consent was taken. Investigations were done regarding the fitness of the patient for anesthesia, and included complete hemogram, random blood sugar, urea, creatinine, electrolytes, electrocardiogram and a chest radiograph. This study was approved by the hospital ethical committee. Laparoscopic procedure has been performed mostly by total extraperitoneal approach (TEP), and some by transabdominal approach (TAPP). In TEP, we have made some modifications, required for the contemplation of the procedures in our limited setup: 1. the extraperitoneal dissection was by direct telescopic dissection under vision. No commercially available or indigenous balloons were used. 2. Direct hernias were reduced, whereas indirect hernias were ligated proximally and distal sac was left by itself. 3. Meshes were not fixed; they were left in place before deflating the preperitoneal space, in the belief that the pressure of the peritoneum holds the mesh in place. No tacks were used, because they are expensive. 4. The meshes used were flat heavyweight polypropylene meshes (10×15cm), no 3-D or lightweight meshes were used. Patients were operated under general anesthesia. Any conversions from TEP to TAPP and from laparoscopic to open repair were recorded with the specific reason for conversion.

All the patients received test dose of ceftriaxone preoperatively, and at induction, 1 gm of inj. ceftriaxone was administered intravenously, and then repeated 8 and 16 h postoperatively. All patients were advised to void urine just before surgery, catheterization was not done. After the surgery, a standard analgesic regimen was administered (intramuscular diclofenac sodium 75mg 8hrly for 24hrs) followed by tab. diclofenac sodium 50mg on demand for pain relief. Oral cefixime was continued for 5 days. Intraoperatively, operative time and intraoperative complications such as vascular, nerve, or vas deferens injury; peritoneal breach; and pneumoperitoneum were noted. The anaesthetist in charge of each case noted the operation time from the skin incision to the application of the last stitch. Postoperatively, hematoma, seroma, subcutaneous emphysema, wound infection, and early recurrence were noted. A visual analogue scale (VAS) pain scoring system was used to monitor the postoperative pain at 12, 24 and 48 hours. The patients were asked to complete VAS score from 0 (no pain) to 10 (intolerable pain) on 12, 24 and 48 hours in the postoperative period, after explanation by an independent assessor who was unaware of the procedure performed. Feeding was resumed soon after full regain of consciousness. Presence of any surgical infections was noted and patients were assessed by independent surgeons for discharge considering diet, ambulation and requirement of oral analgesics if any. All the patients were encouraged to return to work, when they felt comfortable. All patients received similar instructions to return to normal activity and were requested to keep a diary of the date of resumption of full daily activities. Patients were then reviewed at outpatient department at 1, 2, 4 and 12 weeks after surgery and presence of recurrence or any complications were noted. Those with problems were followed up for a longer period as far as possible. Cosmetic outcome was analyzed at 12 weeks of follow-up using the patient's satisfaction on the surgical procedure and the cosmetic results, using a numeric rating scale, ranging from worst outcome (0 points) to best outcome (10 points). All the data was entered in computer and analysis was done manually using SPSS version 15. Results are presented as Mean ± SD and median where appropriate.

## Results

A total of 50 patients were included in the study. They were predominantly males (48 males, 2 females) and their age ranged from 18 to 71 years with 34 being median age at presentation. Maximum patients belonged to age group 31-40 years, followed by 41-50 years. There were 5(10%) bilateral, 35 (70%) unilateral right, and 10 (20%) unilateral left hernias. The majority of hernias were indirect (42, 88%) and only 6 hernias (12%) were direct (Table 1). One of the patients had a direct hernia on the right side and indirect on the left side. Two patients had pantaloon hernia. Two of the indirect hernias were associated with undescended testis. Four of them (8%) had co-morbid conditions (diabetes mellitus in 2, hypertension in 1, and asthma in 1). One had an associated umbilical hernia (TAPP), and another had symptomatic gallstones (TEP with laparoscopic cholecystectomy), another had a recurrent indirect inguinal hernia (TEP). In 41 patients, TEP repair was attempted. Of these, 2 (4.8%) repairs were converted to TAPP and 2 to open mesh repair (4.8%) (Table 2). In 2 patients with undescended testis, orchidectomy was done in addition to hernia repair, one via TAPP, and the other via TEP. In 9 patients, TAPP was started primarily. This group included 4 patients with bilateral indirect hernias, one with undescended testis, and one with the associated umbilical hernia. Among 5 patients with bilateral hernias, 4 underwent TAPP and one underwent TEP

**Table 1:** Characteristics of the patients

Characteristics	n	%/Range
Total no of patients	50	
Males	48	96
Females	2	4
Median age (years)	34	range 18-71 years
Median duration of presentation (months)	16.5	range 1-180 months
Side		
Right	35	70
Left	10	20
Bilateral	5	10
Type of hernia		
Direct	6	12
Indirect	42	84
Both	2	4

**Table 2:** Type of repair performed

Type of repair performed	n	%
TEP	37	74
TEP-unilateral	36	72
TEP-bilateral	1	2
TAPP	9	18
TAPP-unilateral	5	10
TAPP-bilateral	4	8
TEP to TAPP	2	4
TEP to open	2	4
Total	50	100

TEP: total extra-peritoneal; TAPP: transabdominal pre-peritoneal

The most common problem in TEP was and peritoneal breech leading into the leakage of CO2 into the peritoneal cavity, (13 patients, 31.7%) and subcutaneous emphysema in 12 patients (29.2%) (Table 3). A Veress needle was inserted above the umbilicus to decompress the intraperitoneal CO2 in 9 (21.9%). In one patient, it was uncontrollable, and hence was converted to open repair. The other complications noted were wound infection in 3(6%); vas injury in 2(4%); recurrence in 1(2%); seroma in 2 (4%); urinary retention in 2(4%) and bleeding due to inferior epigastric artery injury in 3 patients (6%) and vascular adhered sac in 1 patient (2%) because of long standing hernia with dense extra peritoneal adhesions which could not be managed laparoscopically, this was converted to open repair. Pneumoscrotum occurred in 10 (24.3%) patients intraoperatively, which was managed by squeezing of the air back into the working space during desufflation; it did not persist in the postoperative period. All of subcutaneous emphysema except one resolved in 24 hours; only one case took 48 hours to resolve. Two patients had to be converted to open repair, one due to densely adhered vascular sac which started to bleed profusely, and another due to pneumoperitoneum which could not be controlled by Veress needle, and it severely limited the operative space. These occurred in the first ten of the series. Two cases required conversion from TEP repair to TAPP repair, one due to persistent pneumoperitoneum, due to a large hole in the sac and other due to inferior epigastric artery injury.

**Table 3:** Complications of the techniques

Complications (intraoperative and postoperative)	n	%
Peritoneal breech	13	31.7
Subcutaneous emphysema	12	29.2
Pneumoscrotum	10	24.3
Bleeding	3	6
Wound infection	3	6
Vas injury	2	4
Urinary retention	2	4
Seroma	2	4
Conversion to open	2	4
Recurrence	1	2

The operative time ranged from 50 to 190 min (Table 4). The average operative time for unilateral TEP was 87.21±30.48 minutes (range 50-185 minutes), for bilateral TEP 120 minutes; whereas for unilateral TAPP was 95.4±12.34 min and for bilateral TAPP was 140±46.03 minutes (range 75-190 minutes). The median total hospital stay was 4 days (range -32 days), the mean postoperative stay was .38±3.14 days (range 2-23 days), average time taken for full ambulation postoperatively was 2.05±1.39 days (range 1-10 days), and median time taken to return for normal activity was 5 days (range 2-50 days) (Table 4).

**Table 4:** Operative and postoperative characteristics of the patients

Parameters	Mean/Median and Standard deviation	Range
Mean operative time for unilateral TEP (min)	87.21&+macr;30.48	50-185
Mean operative time for bilateral TEP (min)	120	NA
Mean operative time for unilateral TAPP (min)	95.4&+macr;12.34	85-130
Mean operative time for bilateral TAPP (min)	140±46.03	75-190
Mean VAS score at 12 hours	6.8±1	4-9
Mean VAS score at 24 hours	5.8±1.3	3-8
Mean VAS score at 48 hours	2.8±1.1	1-6
Mean time taken for full ambulation (days)	2.05±1.39	1-10
Mean postoperative stay (days)	3.38±3.14	2-23
Median total hospital stay (days)	4	3-32
Median time taken to return to normal activity (days)	5	2-50
Mean satisfaction score at 12 weeks ( out of 10)	8.67	3-9.5

Only two patients developed urinary retention. The average VAS score at 12 hours was 6.8±1 (range 4-9) which decreased to 5.8±1.3 (range 3-8) at 24 hours and to 2.8±1.1 (range 1-6) at 48 hours postoperative period. One patient developed recurrence (2%), which was during the hospital stay itself. He had huge bilateral indirect hernias, TAPP was done, and after 3 days he developed abdominal distension with recurrent right sided swelling. Laparoscopy was done, which showed that there was recurrence on both sides; on the left side omentum had gone displacing the mesh laterally, which could be reduced with relative ease; this was repaired by putting an additional mesh on the deep ring, laparoscopically and closing the peritoneum. On the right side, ileal loops had incarcerated, which could not be reduced laparoscopically, so open repair was done. He later developed mesh infection, which later could be controlled with daily dressings. He stayed for 23 postoperative days in the hospital, and took 2 months for his wound to heal.

Patients were followed up for a range of 2 weeks to 10 months (mean 6.43 months). The follow-up rate was 81.23% till the third visit. There was no recurrence in the follow-up period. Two patients in whom TEP was converted to open had neuralgia; one had till 2nd visit, i.e. upto two weeks and one had upto 3rd visit, i.e. 4 weeks. Afterwards they had no such complaints. None of the patients who had laparoscopic repair completed complained of neuralgias. Patients reported a mean satisfaction score of 8.67 (range 3-9.5) at 12 postoperative week.

## Discussion

The first laparoscopic hernia repair was described by Ger [3]. Many studies have shown that laparoscopic repair similar results in terms of recurrence compared with open repair, but with the added advantage of reduced postoperative pain and wound infection, and early return to activity [1, 4–7]. Due to these advantages, the time-tested open hernia repair has been slowly replaced by the laparoscopic method. The current study is the authors′ early experience regarding the technique. Inguinal hernia repair was first started by using the TAPP approach. Initially, TAPP became the commonly performed laparoscopic procedure and a number of studies demonstrated the efficacy of TAPP and its comparable results with open hernia repair [1, 5-6, 8]. Although the laparoscopic repair was a new experience for the author, the author has ventured to perform TEP first, a trend which is not popular worldwide; because it is believed that TEP has a steeper learning curve than TAPP, and many recommend that TAPP should be mastered first before TEP. But due to various advantages as non-violation of the peritoneum, no need of suturing in the absence of tacks which prolongs the operating time, we stepped onto TEP first. The current study highlights the short-term outcomes of laparoscopic repair mostly via TEP in our limited setup.

The operative time for laparoscopic repair looks longer than in other studies. Khoury et al [9] and Chung et al [10] also reported a long operating time for the laparoscopic repair. Lal et al [11] found mean operative time for open repair to be 54±15 minutes whereas 75.72±31.6 minutes for laparoscopic repair which was statistically significant (p11]. Anderson et al observed mean operative time of 59±20 minutes for open repair and 81±27 minutes for laparoscopic repair (p12]. Vidovic et al have even reported a shorter time for the laparoscopic group: 55.7±11.1 minutes for the open group versus 54.4±15.1 minutes for the laparoscopic group [13]. The mean operative time in our study was more as compared to other studies probably due to our early experience. Several authors have examined the learning curve for the TEP repair and found that 80-100 procedures are required for a surgeon to complete the repair in less than one hour [14, 15]. There have been few non-randomized comparative studies that have compared the two techniques, i.e., TAPP vs. TEP [16–20]. The results of these comparative trials have shown that the two techniques are comparable with regard to the complications, such as vascular and visceral injury. However, the development of port site hernia was shown to be higher in the TAPP compared with the TEP technique. Hernia recurrence was also shown to be higher in the TAPP group. Other complications were similar with both techniques. However, serious intra-abdominal complications occurred in the TAPP group? two patients with bowel obstruction and one with severe neuralgia. These complications were not seen with a completely preperitoneal TEP approach. Although the TEP method is technically more difficult, the mean operative time in the TAPP and TEP groups were similar. The mean hospital stay and the time to full recovery were also similar in the TAPP and TEP groups. A gradual shift towards TEP has been observed worldwide because of its advantages of reduced risk of bowel injury, bowel adhesions, and incisional hernia formation.

A shorter recuperation period after laparoscopic repair has been reported in several other comparative studies [21–24]. Wilson et al also found that rehabilitation to normal activity and return to work was shorter in patients receiving laparoscopic repair (median 7 and 10 days, respectively) than Lichtenstein repair (14 and 21 days) (p25]. The postoperative VAS pain score has also found to be less in the laparoscopic group. In a comparison of open repair with TEP repair, Eklund et al found that 5 years postoperatively, 1.9% of patients who had undergone laparoscopic repair continued to report moderate or severe pain at the inguinal region compared with 3.5% of those in the open repair group [26]. The large meta-analyses and the VA Trial confirmed that laparoscopic repair leads to less postoperative pain, a shorter convalescence, and faster return to work [27–30]. Patients in the laparoscopic group also had an early ambulation (mean of 1.89±0.84 days) as compared to open group (mean of 2.43±1.23 days). In our study also, no patients in which laparoscopic repair was completed complained of neuralgia or impaired sensitivity. A conversion rate of up to 3.6% from TEP to open repair has been reported in different series [31, 32]. In this study, the conversion rate is 4%.

Peritoneal breech is one of frequent complications which often happen due to a thin sac, which is often opened while dissecting it from the cord structures. If that happens, CO2 starts leaking into the peritoneal cavity and it further compromises the extra-peritoneal space. In such a situation, a Veress needle should be introduced above the umbilicus to remove the peritoneal CO2. One case had to be converted for to open repair for this reason only. We were not familiar with TAPP at that time; later, when this happened in 2 patients, we converted to TAPP. The 8% to 67% incidence of peritoneal breach has been reported previously also by Lal et al [11]. He also reported pneumoperitoneum in 5 TEP cases, managed in the similar fashion; transient pneumoscrotum was seen in four cases (16%), which resolved within 3 hours, whereas subcutaneous emphysema was seen in six cases (24%) [11]. Andersson et al also required the laparoscopic operation to be converted to an open repair for 1 of the patients in the TEP group because of the bleeding from injured inferior epigastric vessel [12]. We also had to convert one case into open because of the excessive bleeding from the very much adhered sac, leading to obscure anatomical visualization; this was in the initial stage of our study. Later we got similar problem of injuring the inferior epigastric artery bleed which discolored the extraperitoneal space and rendered the operation difficult; we converted the procedure into transabdominal approach with the addition of a 5 mm port and successfully completed the procedure laparoscopically. Inferior epigastric artery injury and vas deferens injury have also been reported in earlier studies [33–35].

In the present study, there was no hematoma or visceral injury. Overall, the complications were within acceptable limits and comparable to other series [1]. Subcutaneous emphysema was another problem encountered, which led to increased CO2 retention, leading to slightly delayed extubation in elderly patients. The incidence of postoperative urinary retention was higher in study conducted by Winslow et al in the TEP group (7.9%) [36]. However in the present study, postoperative urinary retention occurred in 2 (4%) of cases. All the patients were asked to void before surgery as a standard practice, and we believe that preoperative catheterization in laparoscopic repair is not mandatory. Postoperative seromas collection was present in 2(4%) patients, seroma formation occurred in 12% of cases in the study by Lal et al and has been reported in 1% to 20% of the cases of TEP [13, 37]. In our context, this was seen in the first two cases; afterwards we routinely started to keep a suction drain in the preperitoneal space, which virtually eliminated the chance of this complication in further cases. Wilson et al concluded bruising to be more common after open mesh repair, while cord seromas was the most frequently encountered complication of laparoscopic surgery [25]. Hematoma was not seen at all in our laparoscopic cases, again probably due to our policy of regularly keeping the drains. In our study two patients (4%) developed port site infection. Hematomas, major and minor wound infections have also been reported in earlier studies [38, 39].

Heikkinen et al in 5-year outcome of laparoscopic and Lichtenstein hernioplasties found both laparoscopic and Lichtenstein hernioplasties to have a low risk for hernia recurrence if proper mesh size is used [40]. Butters in a 52 months follow-up after tension-free and laparoscopic hernioplasties found recurrence rates to be low and similar [41]. One meta-analysis conducted by Schmidt et al comparing open and laparoscopic hernia repair states a recurrence rate of 2.7% for open repair and 5.5% for laparoscopic repair after a follow-up of 28 months [42]. Liem et al diagnosed recurrences in 21 patients (recurrence rate at 2 years 3.8%; recurrence rate at 4 years 4.9%) in the laparoscopic group and in 43 patients (recurrence rate at 2 years 6.3%; recurrence rate at 4 years 10.0%) in the open surgery group (p = 0.006) [38]. In the present study whereas recurrence occurred in one case that underwent laparoscopic TAPP repair for bilateral indirect hernias (rate 2% over a mean follow-up of about 6.43 months). He had huge bilateral indirect complete inguinoscrotal hernias; in fact, studies regarding the laparoscopic hernia repair have even excluded large scrotal hernias from their study, but we haven′t done so [43]. The probable cause of recurrence was that we did not fix the mesh in this particular patient, and the viscera had slide between the peritoneum and the displaced mesh to enter the deep ring. After this mishap, we routinely fix the mesh with prolene sutures in TAPP; we haven′t used tacks because they are expensive. Still in TEP, we haven′t fixed the mesh, and there is no recurrence after TEP, the intraabdominal pressure presses the mesh into position. The low rate of recurrence in the current study may be attributable to various precautions that we took in the technique like adequate dissection to place a large mesh (10x15cm), proper skeletonization of the cord and use of drain to prevent hematoma or seroma formation which can lift and displace the mesh; these have been found to be causes of recurrences in earlier studies [38–43].

## Conclusion

We hereby conclude that laparoscopic repair of inguinal hernias could be contemplated safely both via totally extra-peritoneal as well as transperitoneal route even in our setup, though we are at the beginning of this transition. The shortcomings of the present study are few: the sample size is small, follow-up period is short, and this is a descriptive study. Nevertheless, this is our initial efforts, and in future, this study may form a basis for further such studies. In starting cases, though we had to convert to open repair due to some causes, in later cases, we have found out that TEP could be safely converted to TAPP if required. We could even manage recurrence after laparoscopic repair laparoscopically in absence of complications. Most of adversaries in TEP have been intraoperative, which could be managed properly, and none of these operative complications affected the long-term outcomes of patients.. On the basis of these early experiences, laparoscopic hernia repair can be concluded to be a good alternative promise to open repair especially in terms of postoperative pain, return to work, and cosmesis. This study also shows that laparoscopic hernia repair can also be attempted via TEP first with good knowledge of the anatomy and precautions to reduce recurrences, even if be during the learning phase of the surgeon. We have analyzed out failures for recurrences, and in coming years, we direly feel that we should strive for no conversion and recurrences at all; and this can be achieved as we become more familiar with the anatomy and the technique; because the more we practice the more we stride towards perfection. In developing country like ours, we have been able to undergo this procedure with our own modifications yet remain logical in our techniques.
